# Supplementary material for: Explaining a series of models by propagating Shapley values
Source: Nat Commun. 2022 Aug 3;13:4512. doi: 10.1038/s41467-022-31384-3 (PMC9349278; doi:10.1038/s41467-022-31384-3)
Supplement: Supplementary file 1 — Supplementary Information [file 41467_2022_31384_MOESM1_ESM.pdf]

Explaining a Series of Models by Propagating Shapley Values

1 Supplementary Methods

1.1 Data Sets

1.1.1 NHANES I

The National Health and Nutrition Examination Survey (NHANES) I [1] is a national longitudinal study conducted on a random sample of individuals from the United States. NHANES I investigates a number of demographics and socioeconomic variables. We utilize the NHANES I Epidemiologic Follow-up Study (NHEFS) which is designed to investigated the relationships between clinical, nutritional, and behavioral factors originally assessed in NHANES I. The NHEFS study comprised a series of follow up studies that trace the cohort (all persons 25-74 years of age who completed a medical examination in NHANES I) and measure additional variables as well as collect death certificates.

1.1.2 NHANES 1999-2014

The National Health and Nutrition Examination Survey (NHANES) continually collects information on subsamples of the civilian noninstitutionalized US population in two-year cycles. We collected the data from these cycles from 1999-2014 yielding a total of eight release cycles. The surveys collect a variety of laboratory, questionnaire, examination, and demographic data. In particular, the features collected do not match across cycles, so we only utilize variables that are consistently collected across cycles.

1.1.3 ROSMAP Alzheimer's Gene Expression

Gene expression data collected from the Religious Orders Study (ROS) and Memory and Aging Project (MAP) [2, 3]. ROS is a longitudinal cohort study of aging and Alzheimer's disease run by Rush University enrolling individuals from religious communities for longitudinal clinical analysis and brain donation. MAP is a longitudinal epidemiologic cohort study of common chronic conditions of aging run by Rush University that aims to complement the ROS study by enrolling individuals with wider life experiences and socioeconomic status. Both studies aim to study aging and risk of Alzheimer's disease. We utilize gene expression data collected using ChIP-seq and predict Alzheimer's disease status of the corresponding patients.

1.1.4 METABRIC Breast Cancer Gene Expression

Gene expression data collected from Molecular Taxonomy of Breast Cancer International Consortium (METABRIC) [4, 5]. METABRIC analyzes genomic and transcriptomic information from a set of 995 breast cancer tumors. Although the original analyses of the transcriptomic information from the tumors are used for a variety of analyses, we purely utilize the transcriptomic information to predict tumor status.

1.1.5 CIFAR

The CIFAR10 data set consists of 32×32 color images with 10 possible classes that are a labeled subset of the 80 million tiny images data set [6]. The mutually classes include airplanes, automobiles, birds, cats, deers, dogs, frogs, horses, ships, and trucks. In particular, the images were collected by colleagues at MIT and NYU with natural images collected on a number of search engines.

1.1.6 MNIST

The MNIST database consists of 28×28 black and white handwritten digits [7]. The digits are size-normalized and centered in a fixed-size image. There are ten possible classes that correspond to the digits $0, \cdots, 9$.

1.1.7 HELOC

The Home Equity Line of Credit (HELOC) data set [8] is an anonymized data set of HELOC applications from real homeowners. A HELOC is a line of credit offered by a bank as a percentage of home equity. The outcome is whether the applicant will repay their HELOC account within two years. Financial institutions use predictions of loan repayment to decide whether applicants qualify for a line of credit. The data set was released as part of a FICO xML Challenge (`https://community.fico.com/s/explainable-machine-learning-challenge`) and can be obtained under appropriate agreement to a data set usage license.

1.2 Experimental Setup

1.2.1 CIFAR Multiple Baseline

Model Hyperparameters: The model explained is a CNN with the following sequence of layers: a convolutional layer with 32 filters of shape 3 by 3 with a ReLU activation, a convolutional layer with 32 filters of shape 3 by 3 with a ReLU activation, a max pooling layer with of size 2 by 2, a dropout layer with 0.25 probability, a convolutional layer with 64 filters of shape 3 by 3 with a ReLU activation, a convolutional layer with 64 filters of shape 3 by 3 with a ReLU activation, a max pooling layer with of size 2 by 2, a dropout layer with 0.25 probability, a dense layer with 512 nodes with ReLU activation, a dropout layer with probability 0.5, a dense output layer with softmax activation. RMSprop with a learning rate of 0.0001 and decay of 1×10^{-6} is used to optimize the network for a categorical cross entropy loss over 100 epochs with batch sizes of 32. The test accuracy achieved by the model is 75.56%.

Experimental setup: In Figure 2 we explain three explicands with black objects: a plane, a horse, and an ostrich. For the single baseline attributions we utilize DeepLIFT with a single black image as the baseline. For multiple baselines we utilize G-DeepSHAP with a baseline distribution of 1000 randomly sampled images from the training data set. The feature attribution plots take the local feature attributions for the softmax output corresponding to the true label. For simple visualization we take the absolute value of the attributions and average across channels to get a grayscale image which we plot after normalizing the attribution values between zero and one. The pixel distributions are the number of pixels in the gray scale version of the explicand image that fell within ten equally sized gray scale value bins. The attribution distribution is the sum of the attribution mass for the pixels in the original image that correspond to each gray scale value bin.

1.2.2 NHANES Multiple Baseline

Model Hyperparameters: The model we explain is an MLP with four hidden layers with 100 nodes each. The hidden layers have ReLU activation functions and dropout layers in between. The final output node is a sigmoid activation trained to minimize binary cross entropy loss to optimize mortality classification. RMSprop with a learning rate of 0.001 is used to optimize the network over 50 epochs with batch sizes of 128. The test ROC achieved by the model is .872.

Experimental setup: In Figure 3a, the explicand is a randomly chosen older male individual from the NHANES data set. In the top force plot we show G-DeepSHAP attributions for the explicand with a baseline distribution of 1000 randomly chosen samples from the training set. In the bottom force plot we show G-DeepSHAP attributions for the same explicand with a baseline distribution of 1000 randomly chosen samples from older (>60 years old) males from the training set. In Figure 3b, the clusters are obtained by k-means clustering (k=8) the training data with only two features: age and gender. In Figure 3c, the explicands are the older male cluster in the training data (n=1137). We show two summary plots where the top are G-DeepSHAP attributions with a baseline distribution of 1000 randomly chosen samples from the training set and the bottom uses the older male cluster as a baseline distribution. In Figure 3d, we perform an ablation test that ablates all explicands in the older male clusters according to either their most positive or most negative local feature attributions. When ablating we impute by the mean feature value in the older male cluster. Then we evaluate the model's prediction across all of the explicands after ablating features one at a time.

1.2.3 Gene set explanations

Model hyperparameters: We train two GBT classifiers (an implementation of gradient boosting trees) to predict our binary phenotypes (Alzheimer's and breast cancer tumor stage) based on transcriptomic data. The classifiers are trained with a learning rate of 0.3, a max tree depth of 6, and automatic heuristic tree construction. We train with a validation set and 10 early stopping rounds. For Alzheimer's classification we achieve a test ROC AUC of 0.959 and for breast cancer tumor stage classification we achieve a test ROC AUC of 0.932.

Experimental setup: The feature attributions for the tree model are obtained using Interventional Tree Explainer [9]. These attributions correspond to the importance of each gene to the log odds of the output phenotypes. In order to explain these attributions in terms of groups we utilize our group rescale rule to propagate the gene attributions to pathway attributions. For Alzheimer's we fix the baseline distribution to be the training data set and for breast cancer which has more samples we fix a baseline distribution of 100 random samples from the training set for breast cancer.

1.2.4 NHANES Loss explanations

Model hyperparameters: We train an GBT classifier (an implementation of gradient boosting trees) to predict our mortality based on epidemiological features. The classifier is trained with a learning rate of 0.3, a max tree depth of 6, and automatic heuristic tree construction. We train with a validation set and 10 early stopping rounds. For the weight-shifted test set we achieve an ROC AUC of 0.860 and for the non-shifted test set we achieve an ROC AUC of 0.868.

Experimental setup: In Figure 5b, we generate output feature attributions using Interventional Tree Explainer and use G-DeepSHAP's generalized rescale rule to explain the loss in addition to the output. The loss and output attributions are explained with respect to the same baseline distribution of 1000 random samples from the training set. The loss attributions for positive labelled explicands and negative labelled explicands are very different, leading us to plot them as separate dependence plots. In Figure 5c, we generate output and loss feature attributions as before. For the ablation, we do a simplified univariate ablation where we impute the blood loss to the mean of the baseline distribution for samples selected based on largest loss attributions. In Figure 5d, we perform an ablation test that ablates 1000 explicands from the training set according to either their loss or output local feature attributions. When ablating we impute by the mean value of a given feature in the explicands. Then we evaluate the model's prediction across all of the explicands after ablating features one at a time. Each model agnostic approach has a number of samples parameter which corresponds to the number of coalitions for IME/KernelSHAP and the size of the neighborhood in LIME. We set this parameter to 1000 for LIME and use the default settings for IME and KernelSHAP.

1.2.5 MNIST Feature Extraction

Model hyperparameters: We train a CNN model to classify all digits in MNIST. The CNN model consists of a convolutional layer with 32 filters of size 3 by 3 with ReLU activation, a max pooling layer with pools of size 2 by 2, a convolutional layer with 64 filters of size 3 by 3 with ReLU activation, a max pooling layer with pools of size 2 by 2, a dense layer with 100 nodes and ReLU activation, and the dense output layer with 10 nodes and softmax activation. We utilize categorical cross-entropy loss, an Adam optimizer with learning rate 0.001, and train for 10 epochs. Then, in order to utilize the model to to extract higher level features from raw MNIST images, we remove the final output layer. The GBT model we train to predict zeros using the MNIST features has a max tree depth of 5, a learning rate of 0.5, and a binary logistic objective. This model achieves a test accuracy of 0.998 for predicting zeros.

Experimental setup: In this experiment, we train a CNN model and use it to extract features that are fed into an GBT model. In Figure 6a, we show the feature attributions for G-DeepSHAP and three model-agnostic approaches. All models utilize the same baseline distribution of 100 random images to explain the five images we selected. In Figure 6b we report the runtime of these feature attribution approaches, and the ablation of the top 10% of features. In order to ablate the top ten positive (or negative) features, we simply select the pixels with the largest positive (or negative) attribution in the five explicands and impute them with the mean pixels across the baseline distribution. We obtain confidence intervals by repeating this 20 times for different randomly selected sets of five explicands, where we enforce that at least one zero occurs

within the five explicands, because it is the class of interest. Each model agnostic approach has a number of samples parameter which corresponds to the number of coalitions for IME/KernelSHAP and the size of the neighborhood in LIME. We set this parameter to 100,000 for LIME and use the default settings for IME and KernelSHAP.

1.2.6 HELOC Stacked Generalization

Model hyperparameters: In this experiment we train two base-learners. One base learner is a GBT classifier that represents a fraud detection model which utilizes the following features: MSinceOldestTradeOpen, MSinceMostRecentTradeOpen, and NumTradesOpeninLast12M. Although this classifer represents a fraud detection model, we train it to predict risk using a learning rate of 0.1, 100 estimators, and a max tree depth of 3. The other base learner is that represents a credit scoring model which utilizes the following features: AverageMInFile, NumSatisfactoryTrades, NumTrades60Ever2DerogPubRec, NumTrades90Ever2DerogPubRec, PercentTradesNeverDelq, MSinceMostRecentDelq, MaxDelq2PublicRecLast12M, MaxDelqEver, and NumTotalTrades. We train the base learner to predict risk using an MLP consisting of two hidden layers with 100 nodes and ReLU activations and an output layer consisting of a single dense node with sigmoid activation. The binary cross-entropy loss function is optimized using stochastic gradient descent and a learning rate of 0.005. The meta learner is a GBT classifier that represents a bank risk prediction model which utilizes the remaining HELOC features in addition to the outputs of the two base learners. The meta learner uses the following hyperparameters: learning rate of 0.1, 100 estimators, and a max tree depth of 3.

Experimental setup: In Figure 7a, we first train a GBT and MLP base-learner on disjoint subsets of features from the training data. Then we generate scores for the training data and append it to the remaining features. The remaining features and consumer scores are used to train a final GBT model. Finally, we evaluate the final GBT on a held out test data set. In Figure 7b, we create explanations for the meta model using interventional Tree Explainer for the GBT. Then in 7c, we use the generalized rescale rule to propagate the attributions back through the base-learners (GBT and MLP) to obtain attributions in the original feature space. Each model agnostic approach has a number of samples parameter which corresponds to the number of coalitions for IME/KernelSHAP and the size of the neighborhood in LIME. We set this parameter to 100 for LIME and use the default settings for IME and KernelSHAP.

1.2.7 NHANES Stacked Generalization

Model hyperparameters: In this experiment we train five base-learners - MLPs. The MLPs consist of two hidden layers with 100 nodes and ReLU activations. The output layer is a single dense node with sigmoid activation. The binary cross-entropy loss function is optimized via stochastic gradient descent with a learning rate of 0.005. Then we train a two meta-models that use the outputs of the MLPs as inputs. The first is a logistic regression model with an L2 penalty and regularization strength of 1. The second is a gradient boosted trees classifier with a learning rate of 0.1, 100 estimators, and a max tree depth of 3.

Experimental setup: In Supplementary Figure 9a, we first train five MLP base-learners on training data. Then we embed held out validation data using the predictions of the five MLP base-learners. This embedded validation data is used to train the logistic regression and gradient boosting trees models. Finally, all models are evaluated on a held out test data set. In Supplementary Figure 9b, we create meta-level explanations using interventional Shapley value attributions for the linear models (average voting and logistic regression) [10], and interventional Tree Explainer for the GBT. Then in 9c, we use the generalized rescale rule to propagate the attributions back through the base-learner MLPs to obtain attributions in the original feature space.

1.3 Feature attribution plots

In this section we describe a number of plotting techniques for conveying information about local feature attributions. These plots were first introduced in [9].

1.3.1 Force plots

Force plots show the feature attributions for a single explicand in terms of how they drive the model's prediction for the explicand away from the average model prediction across the baseline distribution. The width of the bars indicate the feature attribution value with red indicating a positive affect and blue indicating a negative one. The features corresponding to the largest bars are below with their actual values for the explicand.

1.3.2 Dependence plots

Dependence plots show the feature attributions for many explicands for a single feature. Every point corresponds to a single explicand where the x-axis is the value of the feature and the y-axis is the the feature attribution value. The coloring of the points often denotes the value of a separate feature.

1.3.3 Summary plots

Summary plots show the feature attributions for many explicands and multiple features. Summary plots stack multiple subplots plots for each individual feature. For the feature plots, every point corresponds to a single explicand where the x-axis is the feature attribution value and the y-axis is vertical dispersion representing the frequency of samples with a particular feature attribution value. Finally, the color of each point represents the normalized feature value, with red representing a high value and blue representing a low one. Intermediary feature values are interpolations between red and blue.

1.4 Shapley value axioms

The Shapley values satisfy a number of desirable properties in terms of the set function v. It is uniquely defined by three axioms:

- *Efficiency:* The sum of the Shapley values for each player equals the value of the game with the set of all players (the grand coalition):
$$\sum_{i=1}^{m} \phi_i(v) = v(M) - v(\emptyset) \tag{1}$$
- *Monotonicity:* If a player i always increases game v_1's value more than they would company v_2 for all possible remaining sets of players, then i's attribution for v_1 should be greater than or equal to their attribution in v_2:
$$v_1(S \cup i) - v_1(S) \geq v_2(S \cup i) - v_2(S) \forall S \subseteq N \setminus i \implies \phi_i(v_1) \geq \phi_2(v_2) \tag{2}$$
- *Missingness:* Employees i that don't help or hurt the company's profit must have no attribution:
$$v(S \cup i) = v(S) \forall S \subseteq N \setminus i \implies \phi_i(v) = 0 \tag{3}$$

While the above three axioms determine the Shapley values as a unique solution concept for credit allocation, the Shapley values have a number of additional desirable properties:

- *Symmetry:* If two players have the same marginal impact for all subsets, then they should have the same Shapley value:
$$v(S \cup i) = v(S \cup j) \forall S \subseteq N \setminus i, j \implies \phi_i(v) = \phi_j(v) \tag{4}$$
- *Linearity:* The Shapley values for a linear combination of games is equal to the linear combination of Shapley values for each game:
$$\phi_i(v_1 + v_2) = \phi_i(v_1) + \phi_i(v_2) \tag{5}$$
and
$$\phi_i(av) = a\phi_i(v) \tag{6}$$

1.5 Examples of Shapley value estimators that trade bias for speed

L-Shapley and C-Shapley impose a constraint based on neighborhoods nearby features [11]. This constraint depends on spatial correlation assumptions, and enables polynomial runtime in the neighborhood size for C-Shapley and exponential runtime in the neighborhood size for L-Shapley. We do not include comparisons to L-Shapley and C-Shapley because although they are model agnostic, spatial correlation assumptions only make sense for data such as images and text data. Instead, G-DeepSHAP is meant to be more flexible and accommodate tabular data as in Figures 3, 4, 5, and 7.

Two additional methods that rely on assumptions to produce relatively fast, but biased estimates of Shapley values are Deep Approximate Shapley Propagation (DASP) [12] and Shapley Explanation Networks (ShapNets) [13]. DASP produces estimates for baseline shapley in $O(N^2)$ model evaluations, where N is the number of features. This is relatively slow in comparison to DeepLIFT which requires a single backward pass (on the order of a single model evaluation). Ultimately DASP's estimates are still biased, because the uncertainty propagation they rely on requires assumptions that are not quite true; however, they show lower bias relative to interventional Shapley values in comparison to DeepLIFT. ShapNets produce estimates for baseline shapley in a single model evaluation and have no bias for Shallow ShapNets, a variant that only includes a single hidden layer and has bias for Deep ShapNets, a variant that includes multiple hidden layers.

We do not include comparisons to DASP and ShapNets, for three reasons. (1) They require very specific model architectures. DASP requires first and second order moment matching for each layer, which is not known in general. ShapNets require utilization of their specific architecture where each hidden node can only have a very small number of inputs (2 or 3 typically) and Shapley estimates are built into the model. (2) DASP and ShapNets are designed to produce baseline Shapley value estimates and cannot generate interventional Shapley value estimates as in G-DeepSHAP. (3) Our experiments primarily encompass model stacks that include more than just deep models, in which case these techniques cannot be applied since they are specific to deep models.

1.6 Baseline distribution proof for interventional Shapley values

Proof. Define D to be the data distribution, N to be the set of all features, and f to be the model being explained. Additionally, define $\mathcal{X}(x, x', S)$ to return a sample where the features in S are taken from x and the remaining features from x'. Define C to be all combinations of the set $N \setminus \{i\}$ and P to be all permutations of $N \setminus \{i\}$. Starting with the definition of SHAP values for a single feature: $\phi_i(x)$

$$
\begin{aligned}
&= \sum_{S \in C} W(|S|, |N|)(\mathbb{E}_D[f(X)|x_{S\cup\{i\}}] - \mathbb{E}_D[f(X)|x_S]) \\
&= \frac{1}{|P|} \sum_{S \subseteq P} \mathbb{E}_{\mathcal{D}}[f(x)|\text{do}(x_{S\cup\{i\}})] - \mathbb{E}_{\mathcal{D}}[\text{do}(f(x)|x_S)] \\
&= \frac{1}{|P|} \sum_{S \subseteq P} \frac{1}{|D|} \sum_{x' \in D} f(\mathcal{X}(x, x', S \cup \{i\})) - f(\mathcal{X}(x, x', S)) \\
&= \frac{1}{|D|} \sum_{x' \in D} \underbrace{\frac{1}{|P|} \sum_{S \subseteq P} f(\mathcal{X}(x, x', S \cup \{i\})) - f(\mathcal{X}(x, x', S))}_{\text{single baseline SHAP value}}
\end{aligned}
$$

where the second step depends on an interventional conditional expectation [14] which is very close to Random Baseline Shapley in [15]). □

1.7 Generalized rescale rule is exact for linear models

We define a series of models composed of linear functions: $f_k(x) = B^k \cdots B^2 B^1 x$ where $B^i \in \mathbb{R}^{o_i \times m_i}$, $m_1 = m$, and $o_k = 1$. If we define $\hat{\phi}$ to return Interventional Shapley values for linear models ($\phi(f, x^e, x^b) = \beta(x^e - x^b)$ where x^e and x^b are the inputs to the linear model and $f(x) = \beta x$ [10]). Then, the generalized rescale rule

gives:

$$\psi^k = B^k(f_{k-1}(x^e) - f_{k-1}(x^b)) \tag{7}$$

$$\psi^i = B^i(f_{i-1}(x^e) - f_{i-1}(x^b))\big(\psi^{i+1} \oslash (f_i(x^e) - f_i(x^b))\big),\ i \in 1, \cdots, k-1 \tag{8}$$

Therefore,

$$\phi_i(f_k, x^e, x^b) = B^k \cdots B^2 B^1 (x^e - x^b) \tag{9}$$

This coincides with the interventional Shapley values for $f_k(x)$ since the composition of linear models is linear.

2 Supplementary Notes

2.1 Additional CIFAR bias examples

We present additional examples of bias for IME and integrated gradients in Supplementary Figures 1 and 2.

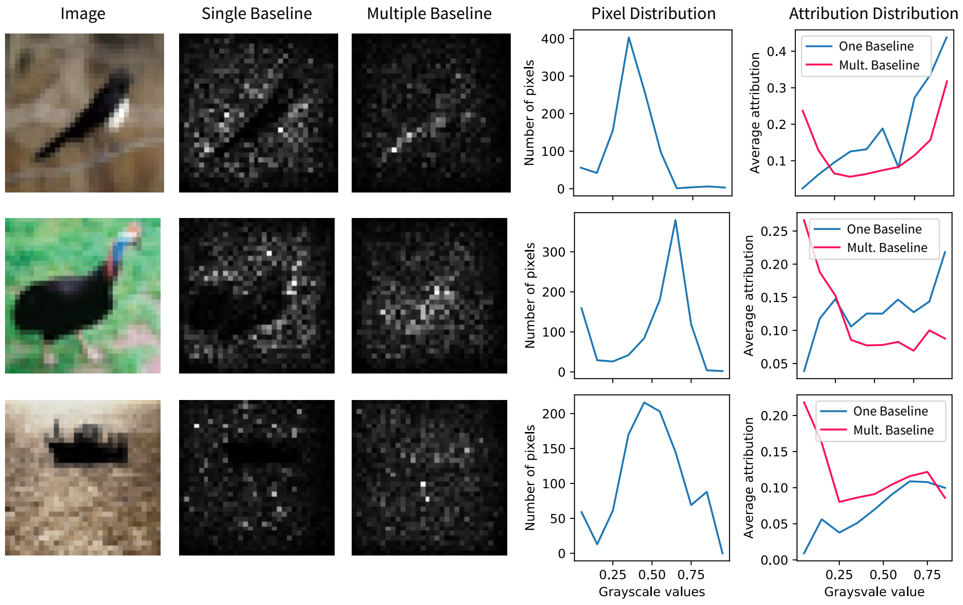

Supplementary Figure 1: Demonstrating bias of a single baseline for IME. The image is the explicand. The attribution plots are the sum of the absolute value of the feature attributions for the three channels of the input image. The pixel distribution is the distribution of pixels in terms of their grayscale values. The attribution distribution is the amount of attribution mass upon a group of pixels binned by their grayscale values.

2.2 Bias for an average baseline

The original formulation of DeepSHAP was equivalent to DeepLIFT with a single average baseline [16]. However, this approach and any approach which depends on a single baseline is susceptible to bias. We demonstrate this in Supplementary Figure 3, where using an average baseline biases the attribution to give low importance to parts of the image which resemble the baseline (dark areas in Delta of Image and Average Baseline).

2.3 Variability due to baselines

In practice, it is standard to estimate interventional Shapley values using a subsample of baselines. In our experiments we typically use 1000 random baseline samples from the general population (the full training

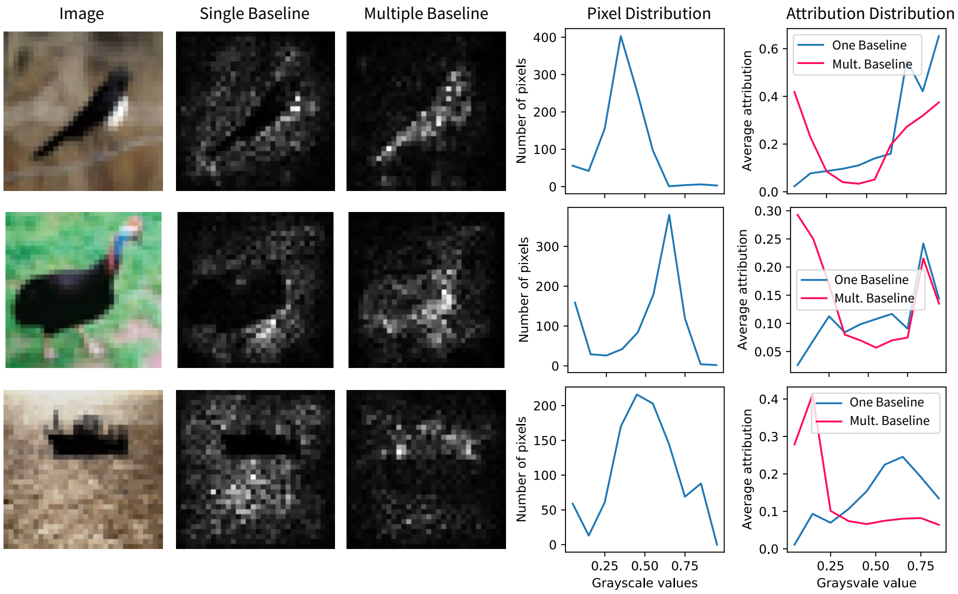

Supplementary Figure 2: Demonstrating bias of a single baseline for integrated/expected gradients. The image is the explicand. The attribution plots are the sum of the absolute value of the feature attributions for the three channels of the input image. The pixel distribution is the distribution of pixels in terms of their grayscale values. The attribution distribution is the amount of attribution mass upon a group of pixels binned by their grayscale values.

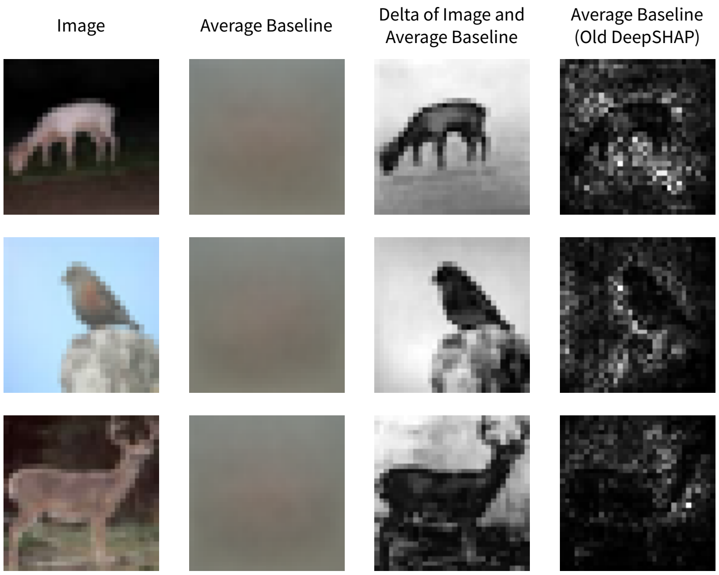

Supplementary Figure 3: Using a single average baseline image (DeepSHAP) leads to biased attributions. The image is the explicand. The average baseline showcases average across 1000 randomly sampled baselines. The Delta of Image and Average Baseline shows the absolute difference between the image and the average baseline summed across channels. Images in this column will have darker pixels in regions where the explicand closely matches the average baseline. The attribution plots are the sum across channels of the absolute value of the feature attributions.

data). Although using a subsample of baselines is faster, it can lead to variability in the resultant attributions depending on the random baselines chosen. In this section, we design an experiment to test how variable our attributions are based on the size of the baseline set. We utilize the NHANES 1994-2014 dataset (Appendix Section 1.1.2), which serves as a suitable testing ground with a large number of samples (35,854) and a large number of features (153).

We first evaluate a few replicates to assess global trends in the attributions for age on mortality risk in Supplementary Figure 4. We find that for both the deep (MLP) and tree (XGB) models, the global attributions are quite consistent with 1000 random baselines. This suggests that there the variability induced by baselines does not change the conclusions drawn from global dependence plots.

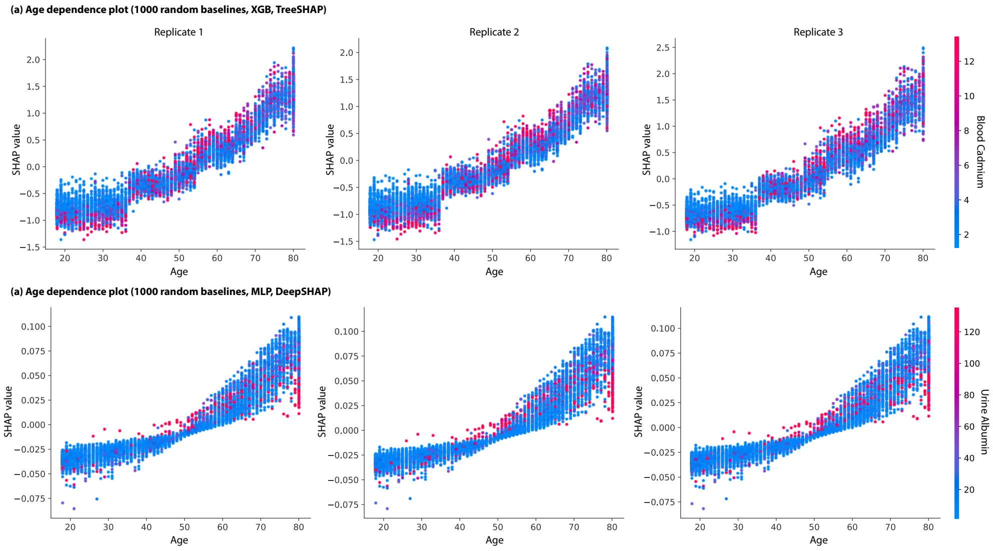

Supplementary Figure 4: Global variability due to baselines. We replicate attributions on the entire test set (n=7034) using three different baseline sets comprising 1000 random samples from the training set. In (a) we show the attributions for a tree (XGB) model and in (b) we show the attributions for a deep (MLP) model. Note that (a) and (b) show dependence plots (Appendix Section 1.3.2).

Next, we evaluate fifty replicates to assess variability in attributions for a single explicand in Supplementary Figure 5. We find that with 1000 random baselines, there is relatively low variance in each feature's attribution value. Furthermore, we see that the attributions converge very quickly with respect to the number of baseline samples. Note that they should be fully deterministic when the number of baselines equals the size of the set from which we draw them (i.e., the training set).

2.4 Probability vs. log-odds explanations

In Supplementary Figure 6 we illustrate the difference between explanations in log-odds versus probability space using attributions obtained from rescaling the log-odds explanations provided by TreeSHAP.

2.5 Additional gene sets

We present attributions aggregated by the Reactome canonical pathway gene set and the Biological Process gene ontology gene set in Supplementary Figure 7.

2.6 Improved predictive performance of feature extraction

In Supplementary Figure 8 we demonstrate the efficacy of deep feature extraction fed into a tree model for MNIST.

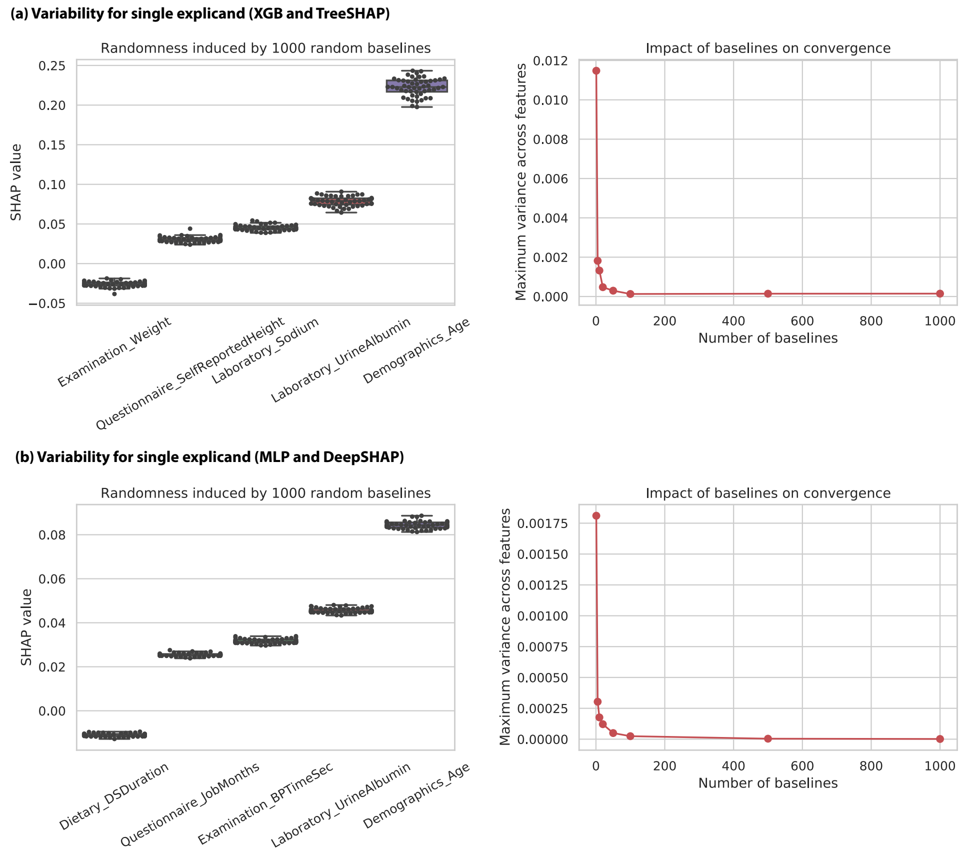

Supplementary Figure 5: Local variability due to baselines. On the left we visualize the attributions for the five features with the highest variance across fifty replicates for a single explicand. Each replicate uses a different randomly chosen set of 1000 baselines. On the right we visualize the attributions' convergence in terms of the maximum variance for each feature across replicates for different numbers of baselines. In (a) we show the attributions for a tree (XGB) model and in (b) we show the attributions for a deep (MLP) model.

2.7 Stacked generalization

We compare five bagged MLP base-learners (feature attributions in Supplementary Figures 10-14) and three meta-learners (average voting, logistic regression, and gradient boosting trees) that use the base-learners' predictions as features for NHANES (1999-2014) mortality prediction with performance in Supplementary Figure 9a. We see that average voting outperforms any individual MLP and is improved upon by a non-uniform weighting scheme (logistic regression). Finally, stacked generalization with a gradient boosted tree meta-model outperforms both linear approaches.

Since our framework enables attributions that satisfy efficiency at each layer, we obtain the importance each meta model assigns to each base-learner (Supplementary Figure 9b), which is much harder to do for model-agnostic methods because it will require separately estimating the importance for each layer. Although the average voting scheme assigns equal importance to each base model, each MLP's predictions are different, leading to the different shapes in the summary plots. In contrast, the logistic regression model downweights MLP0 and MLP3 and primarily relies on MLP2 and MLP4 which achieved the highest performance. The gradient boosting tree model uses the base-learners in a non-linear fashion. For MLP4, high predictions actually decreases the overall prediction of the meta-learner. These meta-level explanations reveal novel insights that explanations in the original feature space would not. Finally, we can also propagate the meta-level explanations back to the original input space and verify that most models give similarly reasonable feature attributions in Figure 7c.

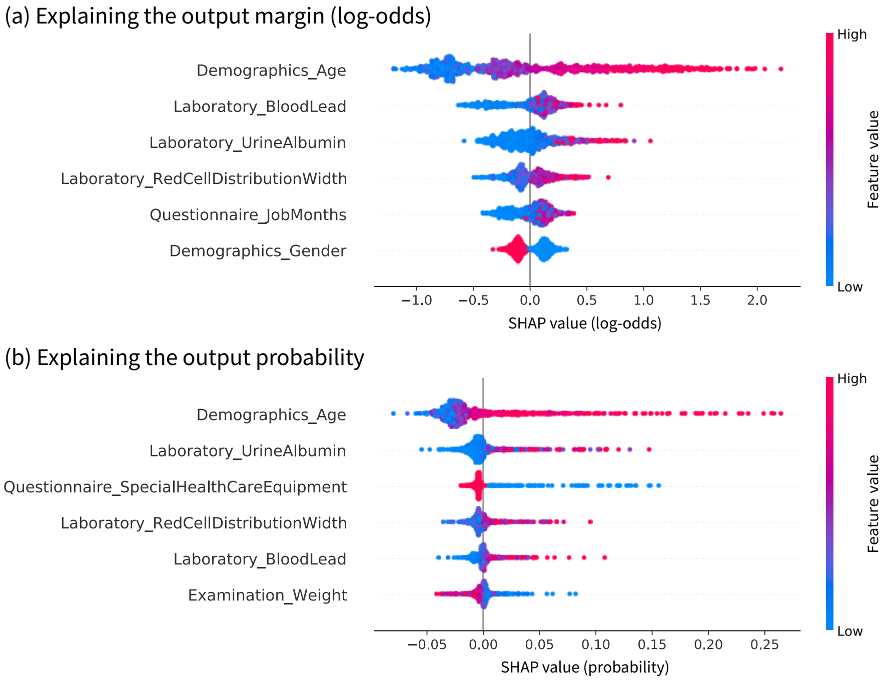

Supplementary Figure 6: The summary plot for the log-odds model output differs to the summary plot for the probability output in terms of ordering of important features. This is to be expected because of the non-linear mapping between log-odds and probability. Often times, it can be useful to communicate scientific findings in terms of the probability output of the model, although the log-odds output is also natural as it is the output margin.

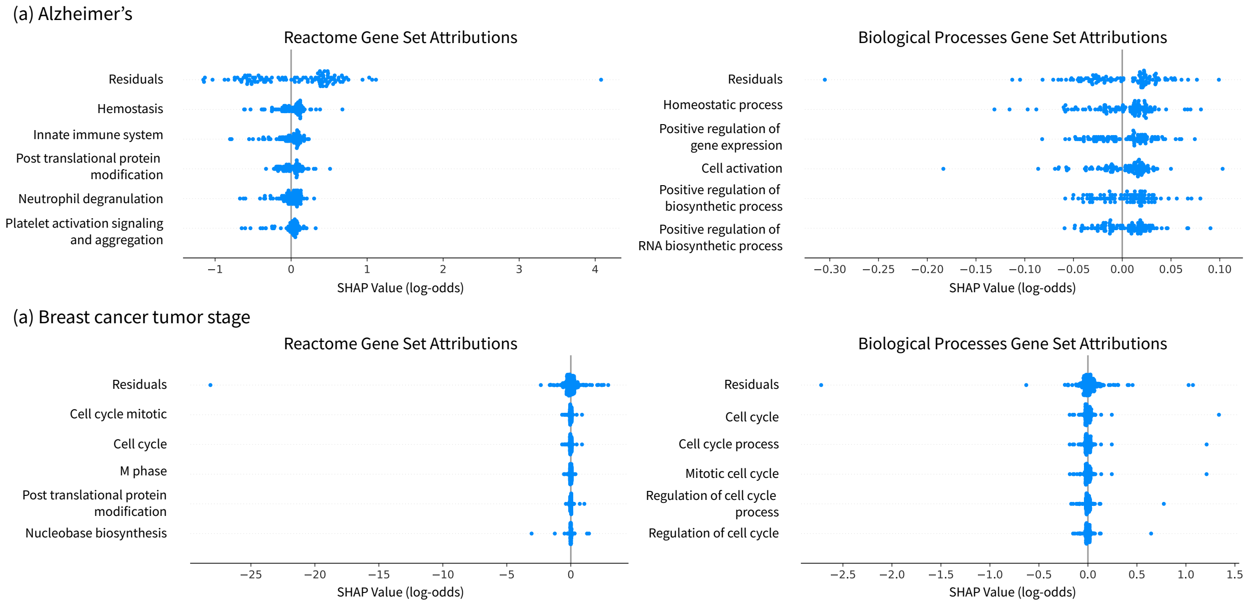

Supplementary Figure 7: Additional gene set attributions. These attributions are based on gene sets from the Reactome canonical pathway gene set and the Biological Process gene ontology gene set. Analogous to the attributions in Figure 4.

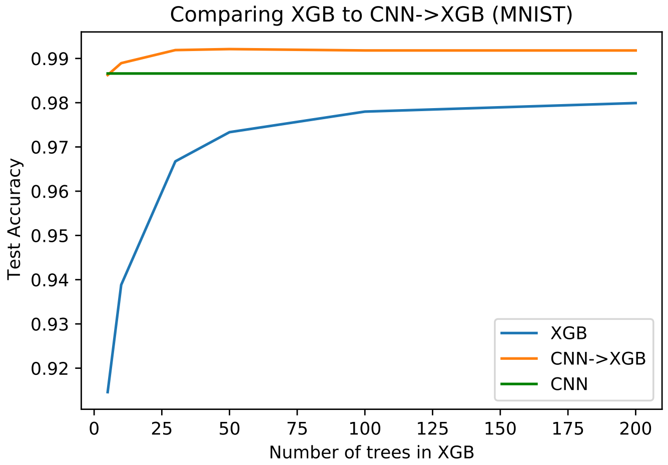

Supplementary Figure 8: Investigating the utility of CNN feature extraction in MNIST. We compare a CNN on the raw digits to an GBT model trained on the raw digits to an GBT model trained to classify digits on the basis of the features extracted by the trained CNN. We vary the number of estimators in GBT to investigate how well underparamterized trees classify digits with different features. Overall, using GBT models with the features extracted from the CNN yield much higher accuracy for trees with the same number of estimators.

2.8 Visualizing interaction effects with G-DeepSHAP

Interaction effects are interactions between the input features that determine the output (e.g., y=x1*x2, where x1 and x2 can be arbitrarily related statistically). Although G-DeepSHAP is not meant to provide estimates of interaction effects, we can still visualize interactions based on vertical dispersion. Vertical dispersion means that the same feature value has different feature importances due to interactions between features. For instance, if two men weigh 180 lbs, then the person who is 5 feet tall probably has a higher mortality risk than the other person who is 6 feet tall. This interaction effect is reflected in Shapley value feature attributions where the importance of weight to mortality risk prediction will be greater for the shorter individual, because he is overweight given his height.

In Supplementary Figure 15, we visualize and qualitatively confirm that meaningful interactions are captured in a multi-layer perceptron explained with DeepSHAP's rescale rule in the NHANES dataset. Firstly, we find that increasing age [17], increasing blood cadmium [18], and decreasing income ratios [19] sensibly correspond to higher mortality predictions. In the age dependence plot we can see that age greatly contributes to mortality risk. Furthermore, we find that age greatly interacts with other features. In particular, we find that although high blood cadmium increases mortality risk for younger individuals, this effect is more drastic among older individuals which is in agreement with evidence that points to an association between blood cadmium levels and Alzheimer's disease mortality among older adults [20]. Similarly, for income ratio, we find that although high income ratios reduce mortality risk in general, the income ratio has a greater impact on mortality risk prediction for older populations. One possible hypothesis for why the effect is more drastic in older populations is that younger individuals can respond to lower incomes by increasing work efforts [21], whereas older individuals may be less flexible.

In terms of the Rescale rule specifically, one mechanism to capture interactions is to encode them in later layers of the network. Then, a single node in a later hidden layer may represent the presence of an interaction. If this interaction is important to the prediction, this node will be important and this importance will be propagated back to the original features that make up the interaction.

2.9 Explanations are robust to predictive performance

One of the main goals of model explanations is to learn about the model's behavior and use it to diagnose unexpected behavior. Interventional Shapley values provide a close description of model behavior and thus

(a) Model performance

Model	MLP0	MLP1	MLP2	MLP3	MLP4	AV	LR	GBT
ROC AUC	0.8207	0.5292	0.8330	0.5042	0.8331	0.8405	0.8425	**0.8444**

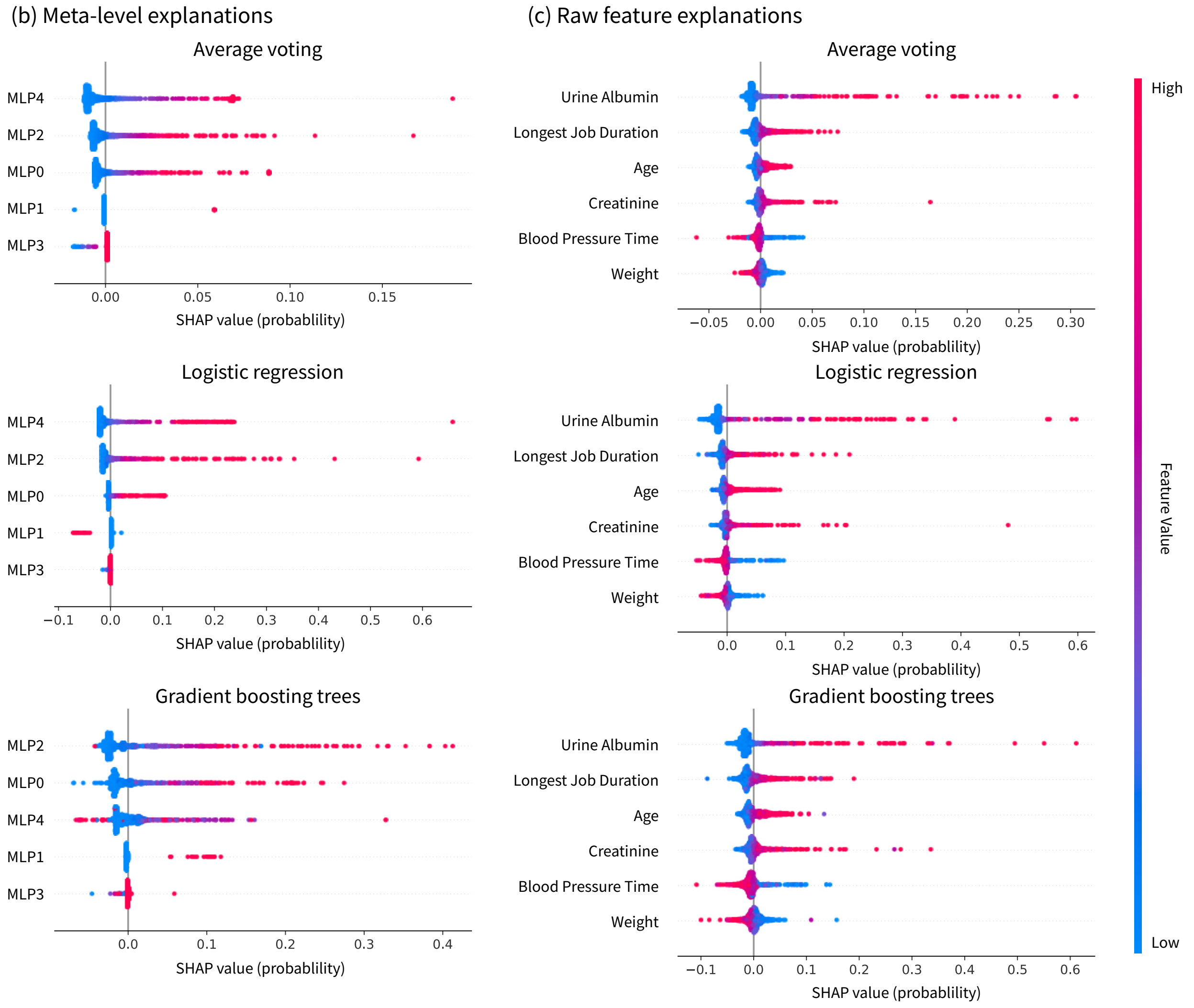

Supplementary Figure 9: Explaining stacked generalization by looking at meta-level and raw feature explanations. (a) The test set performance of the five MLP models and three meta-models that use make predictions based on the MLP models' predictions. (b) Intermediary explanations for the meta-models that assign credit based on which MLP was important to the meta-model's prediction. (c) Raw feature explanations obtained by propagating the credit for each meta-model in (b) to the original feature space. (b) and (c) show summary plots (Appendix Section 1.3.3).

can be used regardless of the quality of the trained model. We design a simple experiment on the NHANES 1999-2014 dataset to illustrate this for two random models: a tree (XGB) model and a deep (MLP) model. Prior to training each model we randomly shuffle the training labels. This results in models with random test performance (ROC ≈ 0.5). Then, we use ablation tests to evaluate the faithfulness of the attributions to the model behavior in Supplementary Figure 16. We find that ablating by the attributions (exact interventional Shapley values for the tree model and approximate for the deep model) drastically changes mean model outputs which indicates they are good descriptions of the model's behavior.

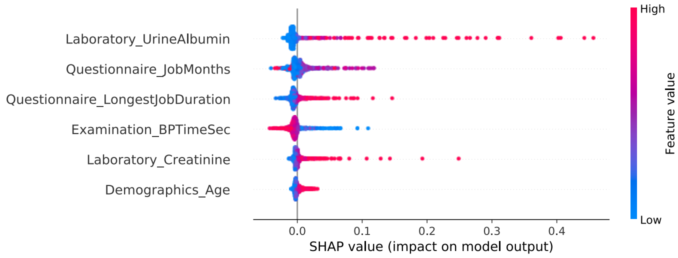

Supplementary Figure 10: Feature attributions for base learner MLP0. The attributions are computed using the rescale rule for the MLP.

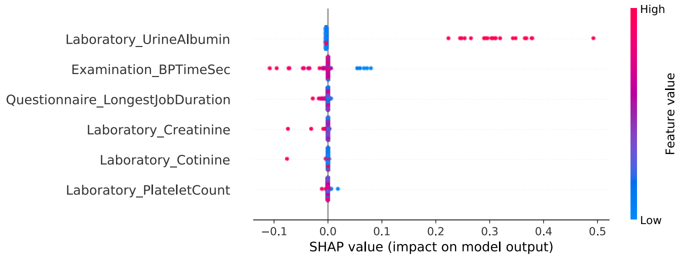

Supplementary Figure 11: Feature attributions for base learner MLP1. The attributions are computed using the rescale rule for the MLP.

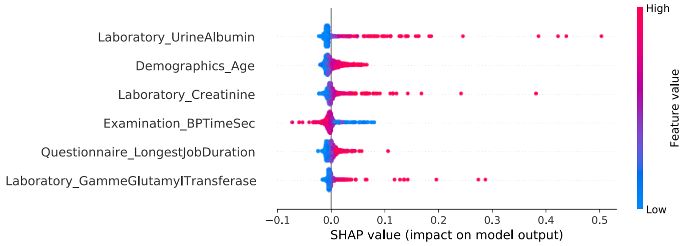

Supplementary Figure 12: Feature attributions for base learner MLP2. The attributions are computed using the rescale rule for the MLP.

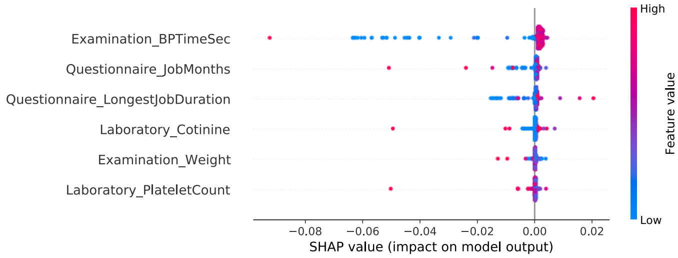

Supplementary Figure 13: Feature attributions for base learner MLP3. The attributions are computed using the rescale rule for the MLP.

2.10 Example of a local ablation

In our ablation tests we primarily report global metrics aggregated over many samples (e.g., mean loss or mean model output). It is worth noting that the global ablation tests are aggregates of local ablations.

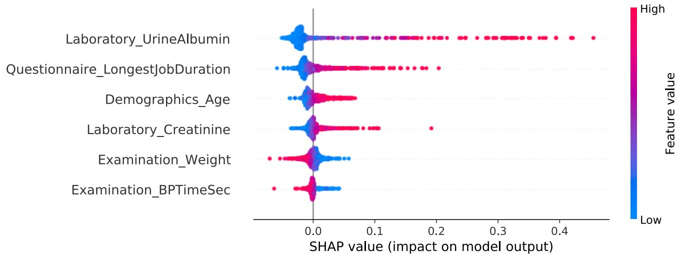

Supplementary Figure 14: Feature attributions for base learner MLP4. The attributions are computed using the rescale rule for the MLP.

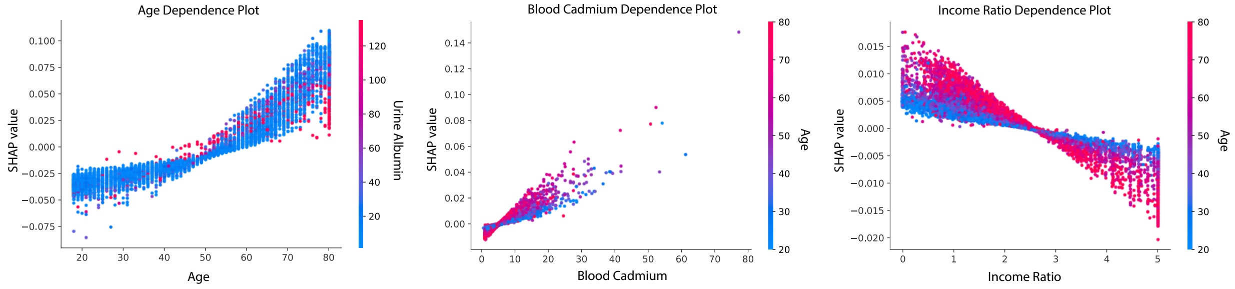

Supplementary Figure 15: Dependence plots for an MLP. We visualize interactions using dependence plots for an MLP with two 128-node hidden layers and dropout layers trained to predict all-cause mortality in the NHANES dataset (test ROC 0.838).

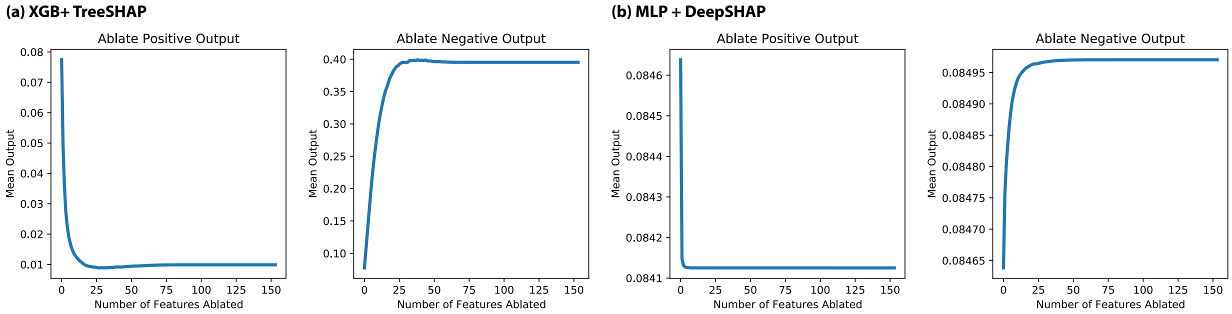

Supplementary Figure 16: Ablation tests for random models. Positive and negative ablation tests based on attributions for (a) a random tree (XGB) model (test ROC 0.416) explained with TreeSHAP and (b) a random deep (MLP) model (test ROC 0.481) explained with G-DeepSHAP. Attributions were computed with 1000 randomly sampled explicands explained using 1000 randomly sampled baselines.

We show an example of one such local ablation for the XGB model (used in Figure 5c and 5d) trained on the NHANES 1999-2014 data and explained using TreeSHAP in Supplementary Figure 17. Local ablation tests serve to assess the quality of the attributions in terms of capturing model behavior for a single sample, whereas global attributions summarize the performance of local ablations across many samples.

2.11 Evaluating convergence of unbiased estimators

In three settings (NHANES loss explanation, MNIST feature extraction, and HELOC model stack) we provide quantitative comparisons to three popular baselines: IME [22], KernelSHAP [16], and LIME [23]. Of these methods, IME and KernelSHAP are known to be unbiased stochastic estimators of the interventional

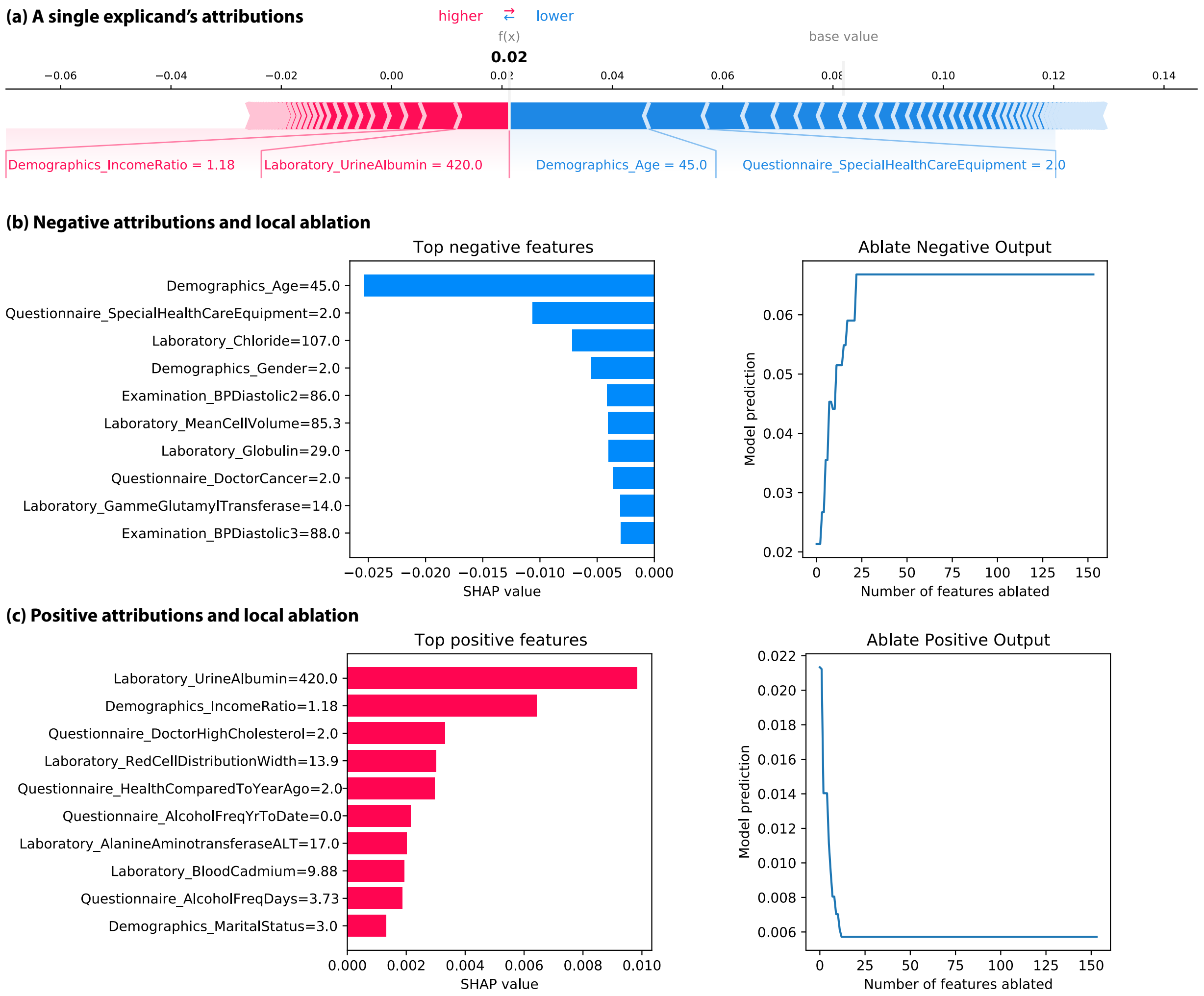

Supplementary Figure 17: Local ablation for a single explicand. Averaging over many of these local ablations gives us the global ablation tests we show throughout the paper. (a) Force plot visualizing the attributions for a single explicand. The axis reports the SHAP value which is in units of the model's output probability. The bars represent each feature's attribution for the explicand (negative in blue and positive in red) and the bottom text denotes the feature name and value. The base value is the average probability across all baselines and $f(x)$ is the predicted probability for the explicand being explained. (b) Sorted negative attributions and the results of ablating these features. (c) Sorted positive attributions and the results of ablating these features.

Shapley values [22, 24]. In particular, we use implementations of each approach (SamplingExplainer and KernelExplainer) from the popular SHAP package (`https://github.com/slundberg/shap`). In the main experiments, we use the default parameter settings for each method. However, one important parameter which affects quality of the estimates and runtime is the number of sampled coalitions. This is because both approaches rely on drawing coalitions from the exponential number of coalitions to approximate the interventional Shapley values. As they draw more and more coalitions, the quality of the estimates improve and converge to the true interventional Shapley values; however, both approaches require evaluating the model on each sampled coalition which can be extremely expensive. In fact, to explain a single explicand to convergence, these approaches may call for tens or hundreds of thousands of model evaluations.

In order to visualize the variability in these estimators, we report the convergence of IME and KernelSHAP across our three datasets and stacked models in Supplementary Figures 18a, 19a, and 20a. In general, we

find that the average standard deviation of the attributions exponentially decays with increasing numbers of coalitions/model evaluations. As one might expect, it gets harder to reduce the variance of the estimators as the number of coalitions increases.

To further investigate whether the variance is significant, we perform ablation tests for varying numbers of coalitions. In Supplementary Figures 18b and 18c, we report the ablation results for ten independent runs of the stochastic estimators (IME and KernelSHAP) and compare them to G-DeepSHAP (which is deterministic) for the NHANES loss explanation example (Figure 5). We find that for lower numbers of samples (500 and 2000), there is significant variance in the ablation results. This suggests that the level of variance is high enough to significantly and negatively affect the ordering of important features for local attributions. For 10000 samples, we find that G-DeepSHAP still outperforms both approaches but by a fairly small margin and both approaches have much less variance in their ablation results. Then, we replicate these experiments for the MNIST feature extraction example (Figure 6) and the HELOC stacked model example (Figure 7) and instead report the results for ablating a percentage of the top positive and negative features. We find that for the MNIST example, G-DeepSHAP greatly outperforms KernelSHAP and IME (Supplementary Figure 19b), suggesting that both methods are not converged even with 10000 samples. In the HELOC example, we find that KernelSHAP outperforms G-DeepSHAP with a fairly small number of samples, although G-DeepSHAP still performs fairly strongly and outperforms IME.

Finally, we visualize the runtimes of each approach in Supplementary Figure 21 to show that even with a tiny number of coalitions, IME and KernelSHAP are drastically slower than G-DeepSHAP. Note that although IME appears to have a constant runtime across the number of coalitions, we note that this may be due to running it on a server which parallelizes the model evaluations across many (56) CPUs. Both IME and KernelSHAP have an asymptotic complexity that is linear in the number of coalitions.

In summation, although G-DeepSHAP may produced biased estimates of the interventional Shapley values, we find that in terms of quantitative metrics (ablation tests) the bias is relatively small. In particular, we find that the the high variance of unbiased stochastic estimators can lead to worse performance and drastically longer runtimes.

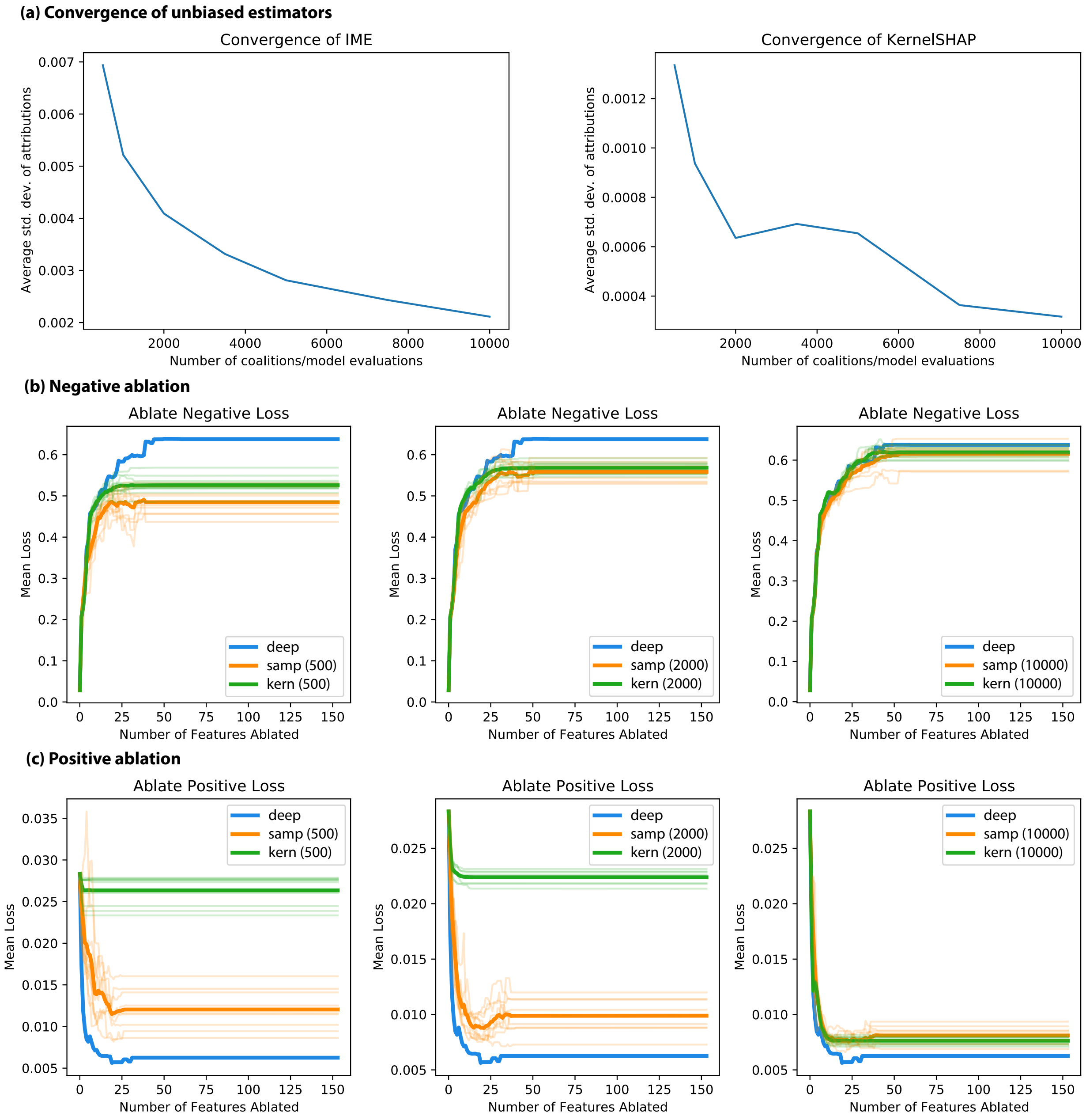

Supplementary Figure 18: Convergence of unbiased stochastic estimators on the NHANES loss explanation example. We explain 10 explicands using 1000 baselines in terms of their loss attributions based on the same model from Figures 5c and 5d. We compute the G-DeepSHAP (deep) attributions once since they are deterministic. Then, we compute the IME (samp) and KernelSHAP (kern) attributions ten times for each number of coalitions to capture their variability. (a) We visualize the average standard deviation per feature across the ten replicates of stochastic estimates. (b) The negative loss ablations. (c) The positive loss ablations. (b) and (c) In the opaque colors we visualize the mean across the ten replicates. In the transparent colors we visualize each of the ten replicates.

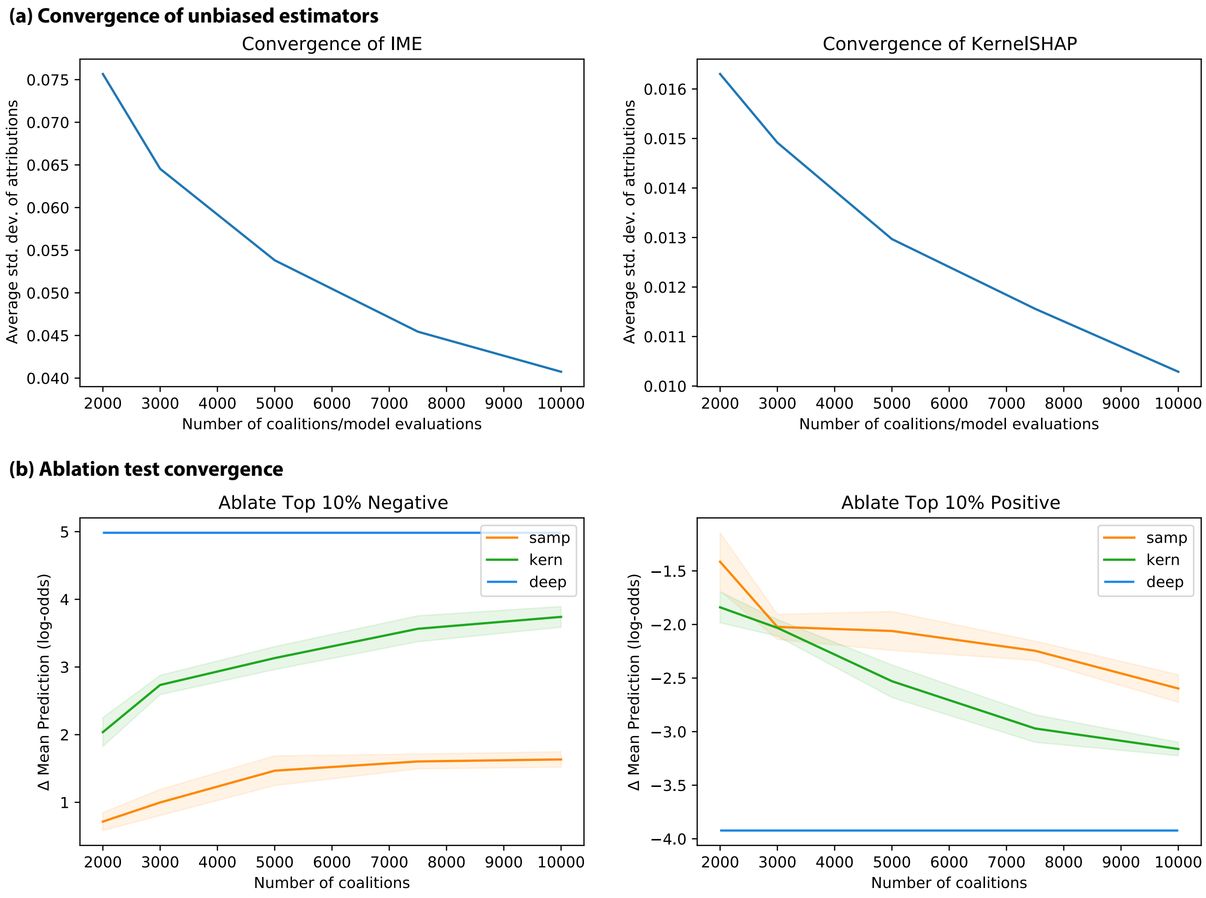

Supplementary Figure 19: Convergence of unbiased stochastic estimators on the MNIST feature extraction example. We explain 10 explicands using 100 baselines in terms of their output attributions based on the same model from Figure 6. We compute the G-DeepSHAP (deep) attributions once since they are deterministic. Then, we compute the IME (samp) and KernelSHAP (kern) attributions ten times for each number of coalitions to capture their variability. (a) We visualize the average standard deviation per feature across the ten replicates of stochastic estimates. (b) Ablating the top 10% negative and positive features. On the left, we visualize ablating the top 10% negative features, for which higher Δs are better. On the right, we visualize ablating the top 10% positive features, for which lower Δs are better. We show the 95% confidence intervals based on the variability of the ablations across replicates.

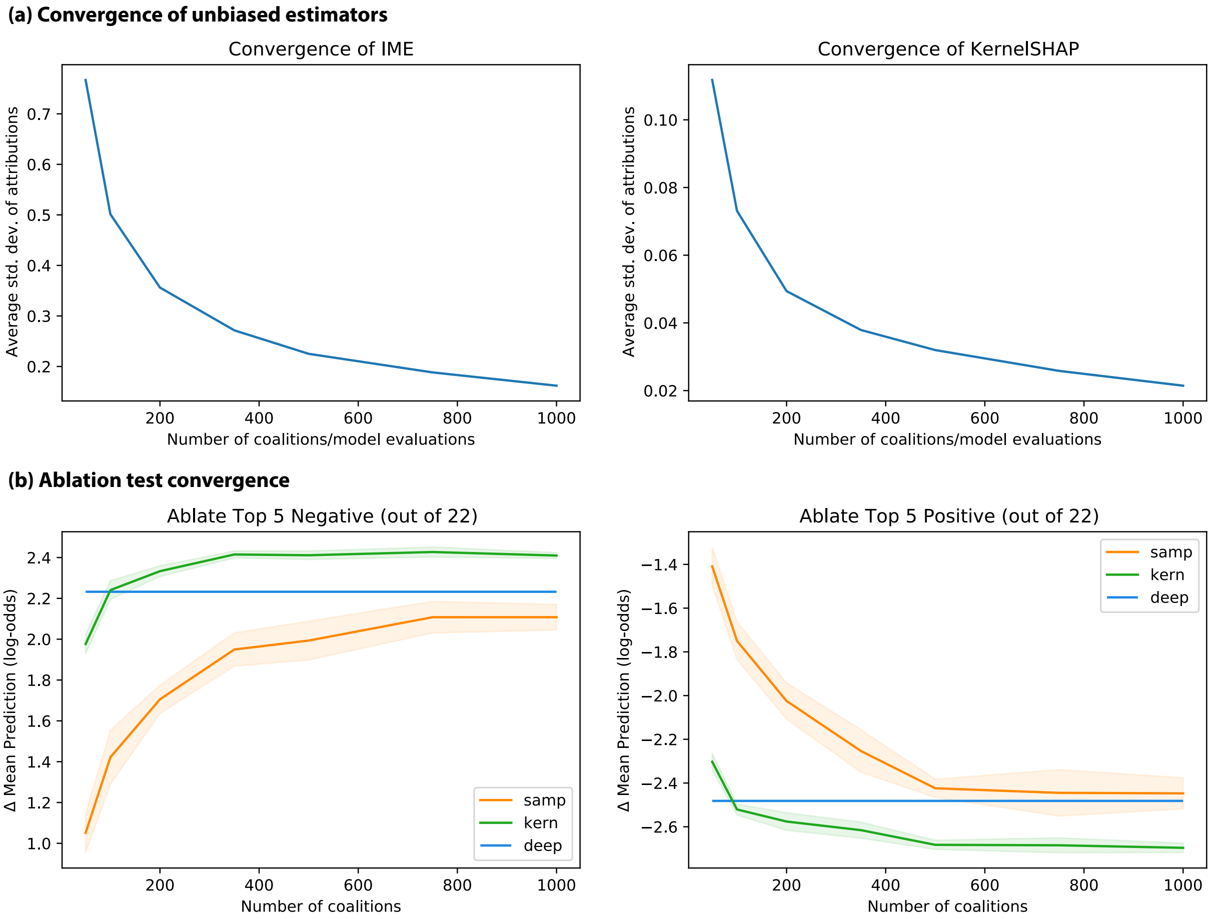

Supplementary Figure 20: Convergence of unbiased stochastic estimators on the HELOC model stack example. We explain 100 explicands using 100 baselines in terms of their output attributions based on the same model from Figure 7. We compute the G-DeepSHAP (deep) attributions once since they are deterministic. Then, we compute the IME (samp) and KernelSHAP (kern) attributions ten times for each number of coalitions to capture their variability. (a) We visualize the average standard deviation per feature across the ten replicates of stochastic estimates. (b) Ablating the top 10% negative and positive features. On the left, we visualize ablating the top 10% negative features, for which higher Δs are better. On the right, we visualize ablating the top 10% positive features, for which lower Δs are better. We show the 95% confidence intervals based on the variability of the ablations across replicates.

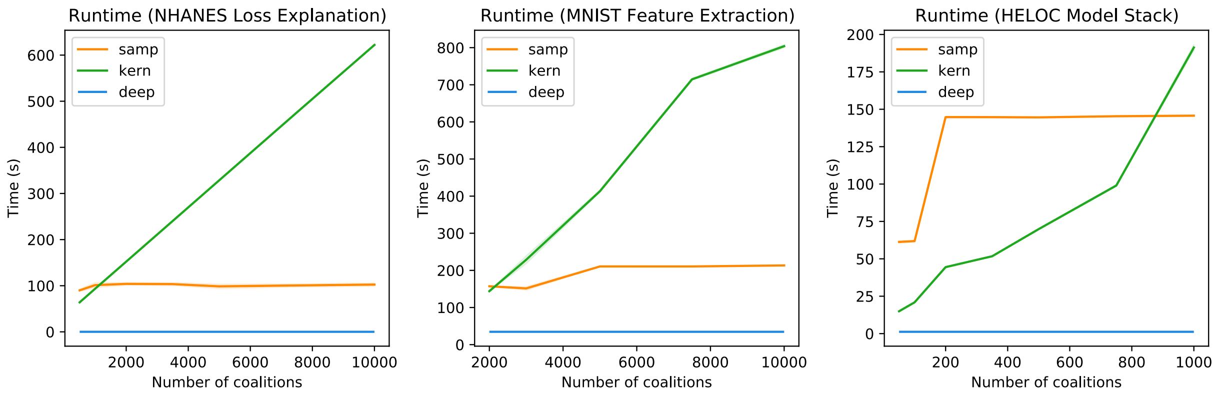

Supplementary Figure 21: Runtimes of approaches from convergence experiments. We report the runtimes of computing explanations based on Supplementary Figures 18, 19, and 20. We also show 95% confidence intervals for IME (samp) and KernelSHAP (kern). For G-DeepSHAP (deep), we only run it once and visualize it's runtime as a horizontal line because it does not depend on the number of coalitions parameter.

3 Supplementary References

1. Cox, C. S. *Plan and operation of the NHANES I Epidemiologic Followup Study, 1992* **35** (National Ctr for Health Statistics, 1998).
2. A Bennett, D., A Schneider, J., Arvanitakis, Z. & S Wilson, R. Overview and findings from the religious orders study. *Current Alzheimer Research* **9,** 628–645 (2012).
3. Bennett, D. A. *et al.* Religious orders study and rush memory and aging project. *Journal of Alzheimer's Disease* **64,** S161–S189 (2018).
4. Curtis, C. *et al.* The genomic and transcriptomic architecture of 2,000 breast tumours reveals novel subgroups. *Nature* **486,** 346–352 (2012).
5. Pereira, B. *et al.* The somatic mutation profiles of 2,433 breast cancers refine their genomic and transcriptomic landscapes. *Nature Communications* **7,** 1–16 (2016).
6. Krizhevsky, A., Hinton, G., *et al. Learning multiple layers of features from tiny images* tech. rep. (Citeseer, 2009).
7. LeCun, Y. The MNIST database of handwritten digits. *http://yann. lecun. com/exdb/mnist/* (1998).
8. *FICO, Xml challenge* `https://community.fico.com/s/explainable-machine-learning-challenge`. [Online; accessed 01-June-2021].
9. Lundberg, S. M. *et al.* Explainable AI for Trees: From Local Explanations to Global Understanding. *CoRR* **abs/1905.04610.** arXiv: `1905.04610`. `http://arxiv.org/abs/1905.04610` (2018).
10. Chen, H., Janizek, J. D., Lundberg, S. & Lee, S.-I. True to the Model or True to the Data? *arXiv preprint arXiv:2006.16234* (2020).
11. Chen, J., Song, L., Wainwright, M. J. & Jordan, M. I. L-shapley and c-shapley: Efficient model interpretation for structured data. *arXiv preprint arXiv:1808.02610* (2018).
12. Ancona, M., Oztireli, C. & Gross, M. *Explaining deep neural networks with a polynomial time algorithm for shapley value approximation* in *International Conference on Machine Learning* (2019), 272–281.
13. Wang, R., Wang, X. & Inouye, D. I. Shapley Explanation Networks. *arXiv preprint arXiv:2104.02297* (2021).
14. Janzing, D., Minorics, L. & Blöbaum, P. Feature relevance quantification in explainable AI: A causality problem. *arXiv preprint arXiv:1910.13413* (2019).
15. Sundararajan, M. & Najmi, A. *The many Shapley values for model explanation* in *Proceedings of the International Conference on Machine Learning* (2020), 513–523.
16. Lundberg, S. M. & Lee, S.-I. *A unified approach to interpreting model predictions* in *Advances in Neural Information Processing Systems* (2017), 4765–4774.
17. Kesteloot, H. & Huang, X. On the relationship between human all-cause mortality and age. *European journal of epidemiology* **18,** 503–511 (2003).
18. Nawrot, T. S. *et al.* Cadmium-related mortality and long-term secular trends in the cadmium body burden of an environmentally exposed population. *Environmental health perspectives* **116,** 1620–1628 (2008).
19. Sabanayagam, C. & Shankar, A. Income is a stronger predictor of mortality than education in a national sample of US adults. *Journal of health, population, and nutrition* **30,** 82 (2012).
20. Min, J.-y. & Min, K.-b. Blood cadmium levels and Alzheimer's disease mortality risk in older US adults. *Environmental Health* **15,** 1–6 (2016).
21. Snyder, S. E. & Evans, W. N. The effect of income on mortality: evidence from the social security notch. *The review of economics and statistics* **88,** 482–495 (2006).
22. Strumbelj, E. & Kononenko, I. An efficient explanation of individual classifications using game theory. *The Journal of Machine Learning Research* **11,** 1–18 (2010).

23. Ribeiro, M. T., Singh, S. & Guestrin, C. *"Why should I trust you?" Explaining the predictions of any classifier* in *Proceedings of the 22nd ACM SIGKDD international conference on knowledge discovery and data mining* (2016), 1135–1144.

24. Covert, I. & Lee, S.-I. *Improving KernelSHAP: Practical Shapley value estimation using linear regression* in *International Conference on Artificial Intelligence and Statistics* (2021), 3457–3465.
